# Both short and long distance migrants use energy-minimizing migration strategies in North American herring gulls

**DOI:** 10.1186/s40462-020-00207-9

**Published:** 2020-06-15

**Authors:** Christine M. Anderson, H. Grant Gilchrist, Robert A. Ronconi, Katherine R. Shlepr, Daniel E. Clark, David A. Fifield, Gregory J. Robertson, Mark L. Mallory

**Affiliations:** 1 Department of Biology, Acadia University, 33 Westwood Ave, Wolfville, NS B4P 2R6 Canada; 2 Wildlife Research Division, Environment and Climate Change Canada, National Wildlife Research Centre, Ottawa, ON K1S 5B6 Canada; 3 Canadian Wildlife Service, Environment and Climate Change Canada, 45 Alderney Dr, Dartmouth, NS B2Y 2N6 Canada; 4 Atlantic Lab for Avian Research, Department of Biology, University of New Brunswick, P.O. Box 4400, 10 Bailey Drive, Fredericton, NB E3B 5A3 Canada; 5 Massachusetts Department of Conservation and Recreation, Division of Water Supply Protection, 485 Ware Road, Belchertown, MA 01007 USA; 6 Wildlife Research Division, Environment and Climate Change Canada, 6 Bruce Street, Mount Pearl, NL A1N 4T3 Canada

**Keywords:** Animal movement, Bird migration, Ecology, Migration strategy, Stopover, Migratory behaviour, Tracking, Telemetry, Generalist

## Abstract

**Background:**

Recent studies have proposed that birds migrating short distances migrate at an overall slower pace, minimizing energy expenditure, while birds migrating long distances minimize time spent on migration to cope with seasonal changes in environmental conditions.

**Methods:**

We evaluated variability in the migration strategies of Herring Gulls (*Larus argentatus*), a generalist species with flexible foraging and flight behaviour. We tracked one population of long distance migrants and three populations of short distance migrants, and compared the directness of their migration routes, their overall migration speed, their travel speed, and their use of stopovers.

**Results:**

Our research revealed that Herring Gulls breeding in the eastern Arctic migrate long distances to spend the winter in the Gulf of Mexico, traveling more than four times farther than gulls from Atlantic Canada during autumn migration. While all populations used indirect routes, the long distance migrants were the least direct. We found that regardless of the distance the population traveled, Herring Gulls migrated at a slower overall migration speed than predicted by Optimal Migration Theory, but the long distance migrants had higher speeds on travel days. While long distance migrants used more stopover days overall, relative to the distance travelled all four populations used a similar number of stopover days.

**Conclusions:**

When taken in context with other studies, we expect that the migration strategies of flexible generalist species like Herring Gulls may be more influenced by habitat and food resources than migration distance.

## Background

Migration strategies, the choices birds make about when to migrate, what routes to take, and when and where to stop, have evolved to maximize fitness in seasonal environments [1]. Migration is a highly dynamic life phase in which birds may travel great distances, often in relatively short periods of time. These journeys can be energetically costly and may entail an elevated risk of mortality [2]. Birds use different migration strategies to minimize these costs and risks; however, a bird’s internal state, its physical capacity for motion, its navigational abilities, and its external environment are perpetually changing and interacting to shape its optimal movement path [3].

Optimal migration theory predicts that animals make trade offs between energy, time, and predation risk when migrating [4, 5]. To achieve high overall migration speeds, birds using a time-minimizing strategy are predicted to use more direct routes, travel long distances per day by flying at higher airspeeds, and/or make fewer stopovers in comparison to birds using an energy minimizing strategy [4]. They should depart with higher fuel loads to permit longer flights [6] and tolerate less optimal wind conditions to avoid delays [7], despite the additional energetic costs these behaviours incur during flight. An alternative approach for some birds may be to minimize energy cost during migration rather than time [4, 5]. Birds using an energy-minimizing strategy are predicted to make more detours away from a direct course to take advantage of the most energetically beneficial conditions for flight or foraging [8]. They should also travel shorter distances per day at lower airspeeds, but conserve energy by optimizing the fuel loads they are carrying [6], or by waiting longer at stopovers for ideal wind conditions to minimize flight costs [9].

The factors that influence migration strategy may vary across the geographic range of a species [10–12]. Long distance migrants are generally predicted to have greater constraints on the timing of their arrival and departure than short distance migrants, and are therefore predicted to minimize time rather than energy during migration [13]. Differences in migration strategy between short and long distance migrants are predicted to be most apparent in autumn for species that are not territorial on their wintering grounds, as there would be no competitive advantage gained by arriving early [14]. Support for the hypothesis that long distance migrants are more time constrained has been demonstrated in shorebirds [15] and passerines [16, 17], and suggested in raptors [18, 19], but some exceptions have been demonstrated [20].

What defines the time constraints on long distance migrants and in what circumstances may they be relaxed? One time constraint for long distance migrants may be the finite amount of time available in a year that birds can budget for migration before the other activities such as molt and breeding are affected [5, 21]. Another major time constraint may be the availability of resources. During autumn migration, there may be increased selection pressure for long-distance migrants to travel before ephemeral food resources are depleted by competitors or diminished by seasonal weather shifts [22, 23], particularly for species specialized on particular prey or habitats [21]. However, migration distance appears to have less of an influence on migratory timing in omnivorous birds, suggesting that dietary plasticity lessens time constraints generated by food scarcity [24].

To test if the hypothesis that long distance migrants are more time constrained applies to flexible generalist species, we compare short and long distance migration strategies of Herring Gulls. Gulls, as omnivorous foraging generalists, can take advantage of many types of food, and therefore can use a variety of terrestrial, freshwater, and marine habitats [25, 26]. Thus, their choice of migratory route may be less constrained by the need to target specific habitats compared to foraging specialists [24]. Gulls are also flight generalists and can use a wide range of flight modes: flapping flight, thermal soaring, ridge soaring, and dynamic soaring [27]. This flexibility allows them to have fewer restrictions on the terrain and weather conditions in which they travel [5]. Gulls also travel with small fuel loads, as they commonly use a fly-and-forage migration strategy and feed along the way [28]. Lastly, gulls have a relatively low risk of predation during migration [29]. Most studies that have investigated how migration distance influences migration strategy have relied on interspecific comparisons [15, 16, 24, 30], where the effect of migration distance is difficult to disentangle from differences in morphology. Migratory strategies, flight behaviour, and related morphological traits (e.g. body size, wing shape) have co-adapted. To address the influence of migration distance on migration strategy, assessing variation in migratory behaviour within species could effectively control for many confounding physiological, morphological, and ecological factors.

We examined the migratory movements of Herring Gulls from four local breeding populations in eastern North America to study the variation in their migration strategies. The rich banding history of this species illustrates that adults breeding in the Great Lakes and on the Atlantic coast of the United States are residents, while adults from Atlantic Canada migrate short distances along the coast, arriving in the northeast United States around November and returning to their breeding sites in April and May [29, 31, 32]. Little is known about the migration of Herring Gulls that breed in the Arctic, but they are presumed to be long distance migrants based on a handful of band recoveries [33]. While banding records have provided information on population level timing and range of migratory movements of some of the Atlantic populations, this study uses tracking technologies to provide new information about how individuals undertake their migratory travels.

Our objectives were: (1) to identify the migration routes of these Herring Gull breeding subpopulations, particularly for the Arctic-breeding Herring Gulls whose wintering distribution was previously unknown; and (2) to test for differences in migration strategies between short and long distance migrants. If long distance migrants are more time constrained than short distance migrants, we predicted that long distance migrants would use more direct travel routes, would travel at higher overall migration speeds in autumn, would make shorter stopovers, and/or would use fewer stopovers per unit of distance [7, 14, 34]. If migration distance does not have a strong influence on the migratory behaviour of Herring Gulls, owing to their ecological flexibility, we expect that both short and long distance migrants would use an energy minimizing strategy. In this case, we would expect each of the Herring Gull populations would follow coastal routes given their preference for aquatic habitats [35], even if this meant detouring from direct routes [28]. For an energy minimizing strategy, we predicted that both long and short distance migrants would have low overall migration speeds, and spend a large proportion of the migration at stopover sites.

## Methods

### Tracking

**Fig. 1:**
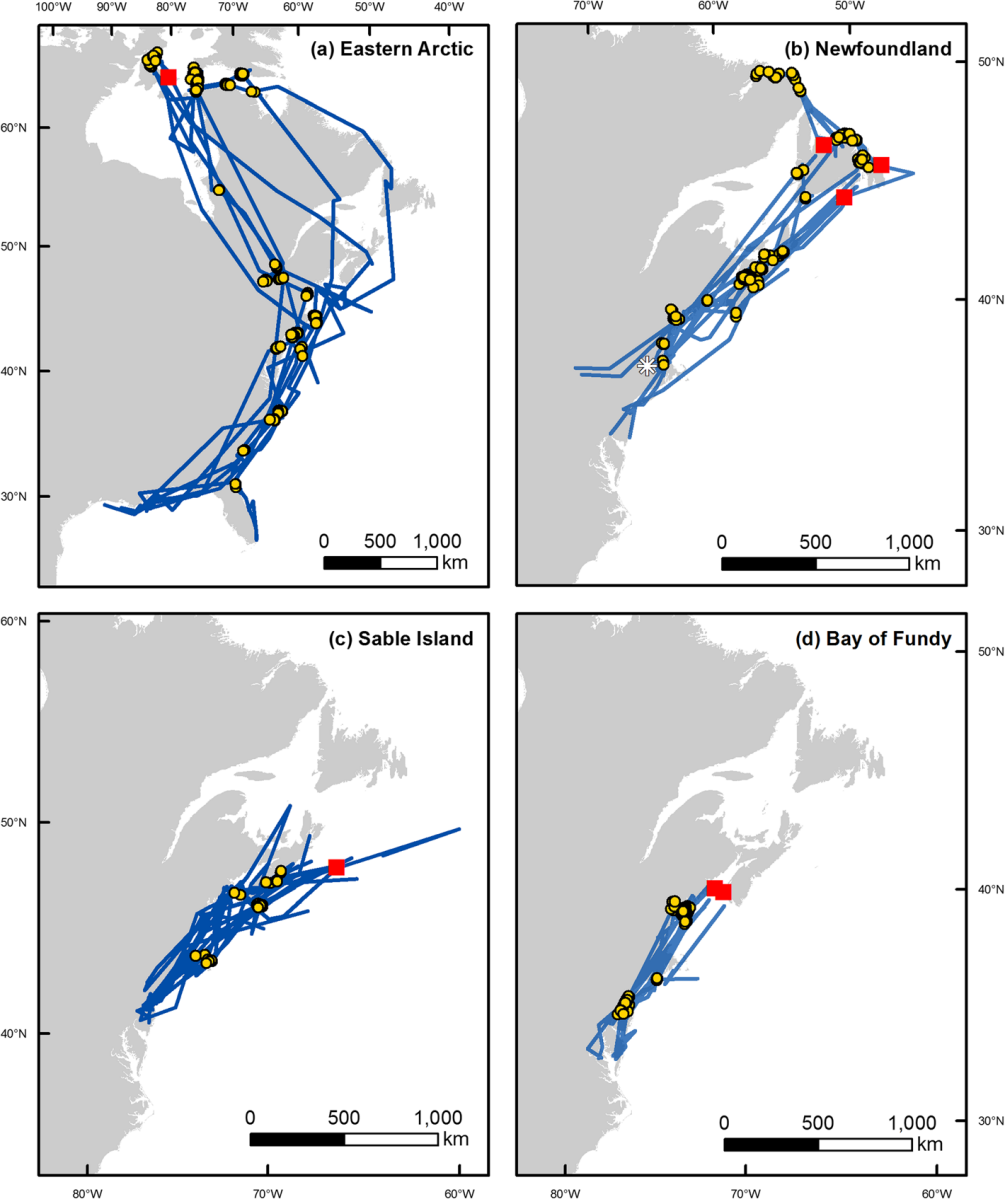
Map of migration routes used by Herring Gulls, predicted by state-space models of tracking data. Breeding colonies are represented by a large red square. All individuals were captured at their breeding colony, except three individuals that bred in Newfoundland were captured during the winter in Massachussets, represented by a white star in panel B. Autumn migration tracks are shown in blue for Herring Gulls breeding in **a**) eastern Arctic (*n* = 8); **b**) Newfoundland (*n* = 12); **c**) Sable Island (*n* = 17); and **d**) the Bay of Fundy (*n* = 11). Stopover days are represented by yellow circles

Between 2008 and 2016, we tracked individuals from four populations of Herring Gulls breeding in eastern Canada (Table 1). Breeding Herring Gulls were captured at one site in the eastern Arctic (Southampton Island, NU, 64.01^0^ N, 81.75^0^ W), two sites in the Bay of Fundy (Kent Island, NB, 44.57^0^ N, 66.75^0^ W; Brier Island, NS, 44.25^0^ N, 66.33^0^ W), on Sable Island, NS (43.92^0^ N, 60.00^0^ W), and at one site in Newfoundland (Witless Bay, 47.26^0^ N, 52.77^0^ W). We also captured wintering Herring Gulls within 75 km of the Quabbin Reservoir in Massachusetts (42.40^0^ N, 72.31^0^ W), which were subsequently tracked to two additional breeding locations in Newfoundland and were assigned to the Newfoundland population in our analyses (Fig. 1).

**Table 1:** Details of tracking device deployment for collecting Herring Gull movement data used in this study

Local Breeding Population	*n* tags deployed	*n* individuals analyzed	*n* migrations analyzed	Deployment Location	Years of Tracking	Model	Duty Cycle	Attachment Method	Capture Method
Eastern Arctic	14	8	8	Southampton Island, NU	2008, 2013–2015	Microwave Telemetry Inc. Solar 18 g PTT-100	10 h on, 24 h off	Leg loop harness [28]	Drop Trap [30]
Newfoundland	9	9	12	Witless Bay, NL	2015–2016	Ecotone Harrier GPS-UHF 15 g	6 locations per day	Leg loop harness	Bow-net [30]
				Within 75 km of Quabbin Reservoir, MA	2009–2013	Microwave Telemetry Inc. Solar 30 g GPS-PTT, Northstar 11.5 g PTT	8 h on, 18 h off	Chest Harness [32]	Net Launcher [29]
Sable Island	8	8	17	Sable Island, NS	2012–2016	Microwave Telemetry Inc. Solar 22 g GPS-PTT	8 locations per day	Leg loop harness	Bow-net
Bay of Fundy	10	8	11	Kent Island, NB	2009–2010,	Microwave Telemetry Inc. Solar 18 g PTT-100	6 h on, 34 h off	Leg loop harness	Drop Trap
				Brier Island, NS	2014–2015	Ecotone Harier-L 13 g GPS-UHF	6 locations per day	Leg loop harness	Drop Trap

Herring Gulls were equipped either with Ecotone devices, which archive global positioning system (GPS) data internally and transmit data to a base station at the breeding site, or with platform terminal transmitters (PTTs), which derive location data from either GPS or Doppler shifts and transmit through the Argos satellite system [36]. Doppler-derived data were collated and processed by Argos, and categorized into four location error classes [36]. Data from GPS were considered to have a fixed location class F, with an error radius of 0 m [37]. Tracking devices weighed 11.5 g to 30 g (< 3% of average Herring Gull body mass; > 800 g, [29]) and were programmed with a variety of duty cycles (Table 1). The effect of tagging on gull behaviour, survival, and reproductive success is generally negligible [38–40], however we suspect that the amount of tension the harnesses were attached with at the Eastern Arctic site may have affected the bird’s survival due to their low return rate in subsequent years.

At the eastern Arctic, Bay of Fundy, Sable Island, and Newfoundland-Witless Bay sites, breeding birds were captured during the incubation period using a self-triggering wire mesh drop trap over their nest [41]. Devices were attached using a leg loop harness, with the transmitter resting on the lower back and secured with loops around the bird’s legs [42]. At the Massachusetts sites, wintering birds were captured using a Coda net launcher hidden under a pickup truck. Bait was placed in front of the net, and the launcher was detonated from inside the truck’s cab [43]. These devices were attached using variations of a chest harness, with the transmitter resting on the upper back, secured with loops around the wings and joined at the chest [44].

### Data processing

We deployed tracking devices on 41 individual Herring Gulls between 2008 and 2016. Of those, we recorded at least one full autumn migration for 33 individuals. Eleven birds were tracked for 2–5 years, giving a total of 48 autumn migration tracks (Table 1).

Tracking data (Argos Doppler data in particular) were recorded at irregular time intervals, and are known often to be less precise than the location error estimates provided by the manufacturer [45]. The data we collected from different sites varied greatly in their sampling frequency, which in turn can strongly influence the interpretation of movement metrics such as distance and directness [46]. To compensate for these issues, we used Bayesian hierarchical switching state-space models to estimate locations at regular 24 h intervals [47, 48]. State-space models estimate the most probable movement path of an individual using two linked components, a process model and an observation model. First, the process model describes the movement path of an individual as a first-difference correlated random walk, switching between two data-driven behavioural states (travelling and foraging) that dictate the distributions of speed and turning angles between locations. Second, the observation model relates the observed data points to the animal’s unobserved location from the process model. The observation model characterizes measurement error by using independently verified data from Vincent et al. [45] to determine the distribution of each Argos location error class. Fitting all individuals within a population using the same state-space model improves the accuracy of location estimates. Additional details about the general parameterization of these models are described in Jonsen et al. [49].

Prior to modelling, we removed duplicate locations and applied a speed filter of 200 km/h. to remove outlier locations from each dataset, enabling more accurate estimates [50, 51]. Based on run length diagnostics of model test-runs [52], we fit state-space models to the dataset using two chains of 400,000 Monte Carlo Markov Chain (MCMC) samples. We discarded the first 50,000 samples as a burn-in, and retained only every 50th sample of the remaining 350,000 samples to reduce autocorrelation. Using the R package CODA [53], we checked the parameter estimates from the remaining 7000 samples for convergence by examining: (1) trace plots of model parameters for good mixing and stationary chains; (2) autocorrelation plots for independence between locations; and (3) density plots, (4) Gelman and Rubin diagnostics [54], and (5) Geweke diagnostics [55] for evidence that posterior distributions were unimodal. We also visually compared the modeled locations to the observed locations. We removed locations that were modeled beyond 1 day of an observed location point, because the state-space model tended to provide biologically unrealistic estimates during large data gaps greater than 1 week [47].

To define when the migrations started and finished, we developed criteria to classify the movement behaviour of each individual as migratory or non-migratory. A position was categorized as a travel day if either: (1) the bird moved more than 0.3° of latitude in the same direction for 2 of 3 days in a sliding window, indicating sustained travel; or (2) the bird moved more than 75 km in a single day, indicating large jumps. Having both of these criteria allow us to exclude days where birds make moderate movements during a stopover, but then return and continue to remain in the stopover area without directed migration movements. This approach has been used by other studies of migratory seabirds; we chose the 90th percentile of change in latitude and distance travelled as our threshold because these criteria categorized the differences in behaviour when visualized and fell within the range of values used in other studies [10, 56, 57]. If the position did not meet either of these criteria, it was categorized as a stopover day. Autumn migration was considered to start on the first day of a period of travel moving beyond a 200 km radius from their breeding colony, and therefore do not include any pre-migratory fueling that may have occurred near the colony. Autumn migration was considered to end when a period of travel finished at a latitude where the individual remained during the winter.

### Migratory strategies

We calculated ten characteristics for each migration track. The orthodromic *migration distance* was calculated only for travel days, as the distance traveled during stopover days varied greatly depending on the length of stopovers and tended not to be directed movement. The *duration* of each migration was the total number of days between the *start date*, when the bird departed the breeding area, and the *end date*, when the bird arrived at it’s wintering latitude. *Directness* was the ratio of an individual’s migration distance to the shortest possible route between their starting and ending location, calculated as the great circle distance (shortest distance between two points on the surface of the earth) using the Vincenty ellipsoid method [58]. The overall *migration speed* was the migration distance divided by the duration of migration, while *travel speed* was the migration distance divided by the number travel days; both are recorded as kilometers per day. We calculated three different aspects of stopover behaviour: *total stopover days* were categorized using the stopover criteria described above, *travel:stopover ratio* was the distance travelled divided by the number of stopover days, and *stopover length* was the number of days spent at each stopover site.

The ten characteristics described above were modeled as the response variables in a set of generalized linear mixed model (GLMM). Breeding population was included as a fixed effect to assess if migration strategy differed between populations. Because gulls have a high degree of individual variation, and some individuals were tracked for 2–5 years, we included individual as a random effect to control for these differences, except in the model for directness where including the random effect resulted in a singular fit. We used Gaussian (migration distance, start date, end date, directness, migration speed, travel speed, travel:stopover ratio), or Poisson (duration, total stopover days, stopover length) distributions, and their respective canonical link functions [59, 60]. Distributional assumptions about the data were checked through graphical analysis of scaled residual plots [61]. We used a likelihood ratio test on these GLMMs against null models with the fixed effect removed, and considered there to be significant differences between populations when *p* < 0.05. We assessed the explanatory power of these models with marginal *R*^2^ (fixed effect only) and conditional *R*^2^ (fixed and random effects [62, 63]). Means are presented ± standard deviation unless otherwise noted.

## Results

### Migratory routes, timing and stopover locations

**Fig. 2:**
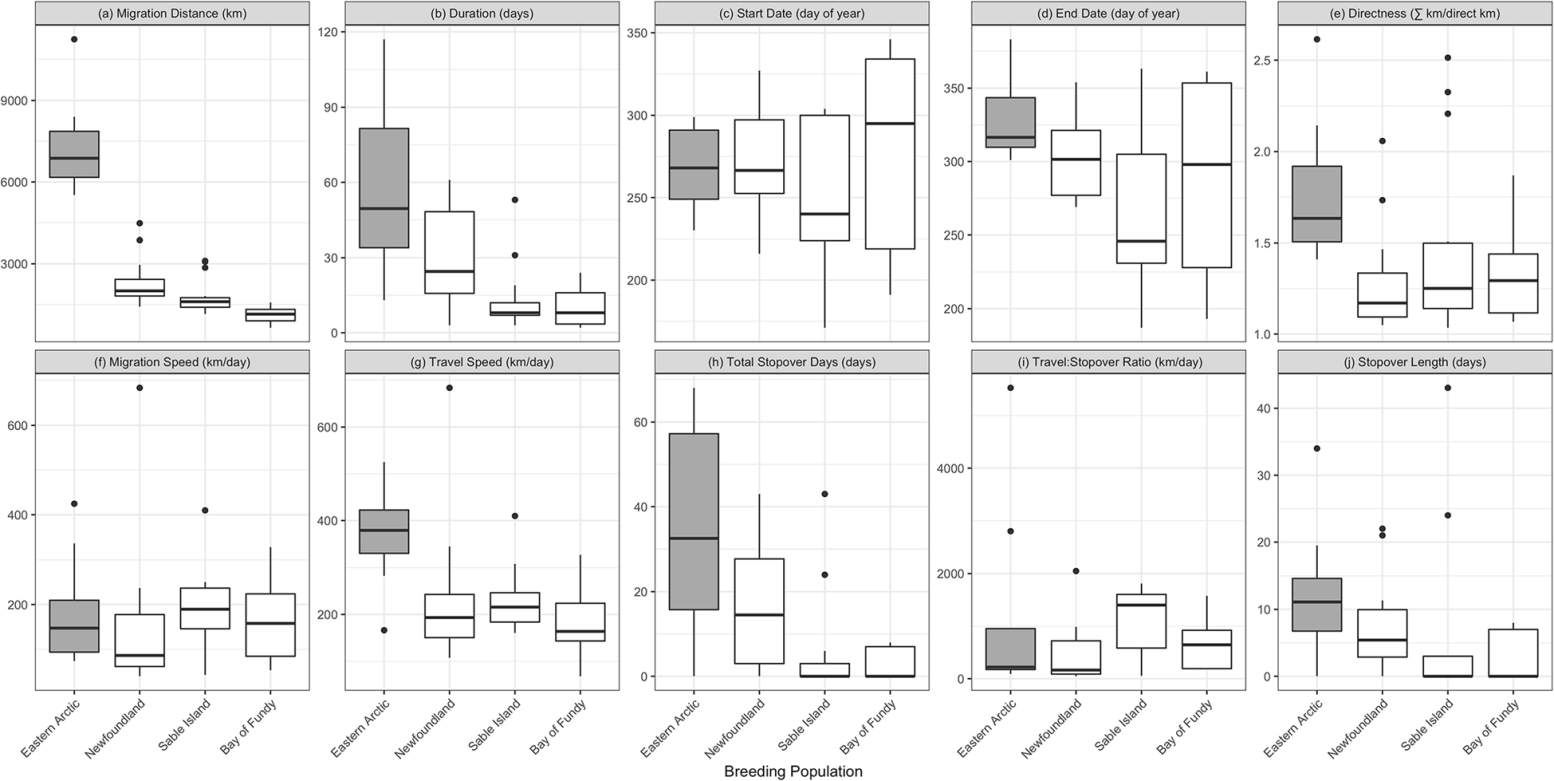
Boxplots illustrating variation in migration characteristics of Herring Gulls in eastern North America. Long distance migrants from the eastern Arctic are shown in grey, and short distance migrants from Newfoundland, Sable Island, and the Bay of Fundy are shown in white. Migration characteristics include **a**) distance, **b**) duration, **c**) start date, **d**) end date, **e**) directness, **f**) migration speed, **g**) travel speed, **h**) stopover days, **i**) travel:stopover ratio, and **j**) stopover length

We analyzed the autumn migrations of 33 Herring Gulls, of which 11 were tracked for 2–5 years, giving a dataset of 48 migration tracks. Herring Gulls from the eastern Arctic traveled an average of four times further than Atlantic Herring Gulls (χ^2^_3_ = 66.79, *p* < 0.01; Fig. 2a). Gulls from the eastern Arctic traveled 7361 ± 1815 km (*n* tracks = 8) during their autumn migrations. By comparison, gulls from Newfoundland migrated 2366 ± 936 km (*n* = 12), gulls from Sable Island migrated 1760 ± 631 km (*n* = 17), and gulls from the Bay of Fundy migrated 1023 ± 374 km (*n* = 11).

Four of the eight of the Herring Gulls breeding in the eastern Arctic made stopovers of 2 weeks or more in Hudson Strait and Foxe Channel at the beginning of their autumn migration. Herring Gulls breeding in the eastern Arctic appeared to use two distinct autumn migration routes. Two individuals migrated using a strictly coastal route, passing east through Hudson Strait, south along the coast of Labrador, and then following the Atlantic coast to their wintering range offshore from Louisiana, Texas, and Mexico [35]. By contrast, six individuals migrated south from their breeding colony through Hudson Bay, made overland crossings of 1588 ± 433 km to the Atlantic Coast between the St. Lawrence River Estuary and Chesapeake Bay, and then followed a coastal route to the same wintering range. Flights from Hudson Bay to the Atlantic coast covered 2000 km or more in 3 days, with only two of these individuals making short stopovers on major freshwater bodies in Québec (St Lawrence River, Lac St. Jean, La Grande River). Migratory stopovers were not concentrated in any particular area, but were spread across the Atlantic coast from Newfoundland to Florida (Fig. 1a). However, four of eight birds made at least one stopover on the Atlantic coast between Maine and Cape Cod, the same area covered by the Atlantic Canada populations’ wintering ranges.

Herring Gulls from the three regions of Atlantic Canada made short distance migrations along the Atlantic coast to their wintering range between coastal Maine and Cape Cod (Fig. 1b-d). All wintering locations were in coastal areas, except two individuals from Newfoundland and one from Sable Island spent part of the winter in the Finger Lakes region of New York State [35]. All stopovers made by Herring Gulls breeding in Atlantic Canada were spread across their migratory route, with no particular stopover site attracting a large proportion of individuals (Fig. 1b-d). Two individuals that bred in Newfoundland moved north from their breeding colony, spending 2–3 weeks at Hamilton Inlet, Labrador, before turning southward for migration.

The duration of fall migration was longer for the birds tracked from the eastern Arctic than for the birds tracked from Atlantic Canada (χ^2^_3_ = 66.79, *p* < 0.01); Herring Gulls from the eastern Arctic took an average of 57 ± 36 days, while gulls from Newfoundland, Sable Island and the Bay of Fundy respectively took 29 ± 19, 13 ± 12, and 10 ± 7 days (Fig. 2b). There was no significant difference in the start date of migration between short and long distance migrants (χ^2^_3_ = 2.69, *p* = 0.44), with an overall mean start date of Sept 22 ± 45 days (*n* = 33, Fig. 2c). However, long distance migrants did have a later end date of their migration compared to short distance migrants (χ^2^_3_ = 9.75, *p* = 0.02); Herring Gulls from the eastern Arctic ended their migrations on Nov 27 ± 32 days. This was comparable to gulls from Newfoundland, which ended their migrations on Oct 30 ± 28 days, but was significantly later than gulls from Sable Island and the Bay of Fundy ended their migrations on, Sept 22 ± 52 days, and Oct 17 ± 67 days (Fig. 2d).

### Migration strategy – directness, speed, and stopover behaviour

Long distance migrants used less direct routes than short distance migrants (χ^2^_3_ = 10.93, *p* = 0.01; Table 2). Birds from the eastern Arctic used routes that were on average 1.79x the length of a direct migration. In comparison, birds from Newfoundland, Sable Island and the Bay of Fundy used routes that were respectively 1.30x, 1.43x, and 1.32x the length of a direct migration (Fig. 2e).

**Table 2:** Parameter estimates (β, 95% confidence intervals) for generalized linear mixed models examining the effects of population on autumn migration characteristics of Herring Gulls. Long distance migrants were tracked from Eastern Arctic (NU; *n* = 8), and short distance migrants were tracked from Newfoundland (NL; *n* = 9), Sable Island (SI; *n* = 8), and the Bay of Fundy (*n* = 8). Individual is included as a random effect in all models except for directness. The intercept is the predicted value for Herring Gulls from Eastern Arctic, which then acts as the reference level for the other parameter estimates, which are relative differences from the intercept. Bold font indicates estimates whose 95% confidence intervals do not cross 0, indicating a significant difference from the Eastern Arctic population, and likelihood ratio test statistics (χ2) where *p* < 0.05

Migration characteristic	Population	β	(95% CI)	Marginal R^2^	Conditional R^2^	χ^2^_3_	p
Migration Distance (km)	Intercept (NU)	6899	(6263, 7536)	0.84	0.94	**67.16**	**<0.01**
	**NL**	**-4652**	**(-5515, -3778)**				
	**SI**	**-5190**	**(-6057, -4324)**				
	**BF**	**-5934**	**(-6822, -5050)**				
Duration (days)	Intercept (NU)	3.9	(3.4, 4.4)	0.47	0.95	**23.33**	**<0.01**
	**NL**	**-0.9**	**(-1.6, -0.2)**				
	**SI**	**-1.5**	**(-2.2, -0.8)**				
	**BF**	**-2.0**	**(-2.8, -1.3)**				
Start Date (day of year)	Intercept (NU)	267	(237, 298)	0.10	0.98	2.69	0.44
	NL	8	(-33, 50)				
	SI	-23	(-65, 20)				
	BF	6	(-36, 49)				
End Date (day of year)	Intercept (NU)	330	(298, 363)	0.25	0.98	**9.77**	**0.02**
	NL	-26	(-70, 18)				
	**SI**	**-72**	**(-117, -27)**				
	**BF**	**-48**	**(-94, -3)**				
Directness (∑ km/direct km)	Intercept (NU	0.59	(0.47, 0.71)	0.17	-	**9.06**	**0.03**
	**NL**	**0.23**	**(0.09, 0.38)**				
	**SI**	**0.18**	**(0.03, 0.32)**				
	**BF**	**0.19**	**(0.04, 0.34)**				
Migration Speed (km/day)	Intercept (NU)	185	(94, 275)	0.00	0.84	0.01	0.99
	NL	-2	(-126,123)				
	SI	-3	(-130,129)				
	BF	1	(-126, 129)				
Travel Speed (km/day)	Intercept (NU)	366	(-290, 441)	0.21	0.82	**9.67**	**0.02**
	**NL**	**-104**	**(-207, -0.3)**				
	**SI**	**-144**	**(-248, -40)**				
	**BF**	**-160**	**(-256, -54)**				
Total Stopover Days (days)	Intercept (NU)	2.9	(1.8, 4.0)	0.37	0.97	**14.61**	**<0.01**
	NL	-0.9	(-2.4, 0.6)				
	**SI**	**-2.6**	**(-4.2, -1.1)**				
	**BF**	**-2.8**	**(-4.6, -1.3)**				
Travel:Stopover Ratio (km/day)	Intercept (NU)	1187	(473, 1902)				
	NL	-622	(-1598, 357)	0.07	0.79	2.48	0.48
	SI	-93	(-1082, 896)				
	BF	-561	(-1563, 441)				
Stopover Length (days)	Intercept (NU	2.4	(1.9, 2.9)				
	NL	-0.7	(-1.4, 0.1)	0.21	0.80	6.16	0.10
	**SI**	**-1.0**	**(-1.8, -0.2)**				
	BF	-0.7	(-1.6, 0.3)				

There was no significant difference between the overall migration speeds of the populations that migrated short and long distances (χ^2^_3_ = 0.01, *p* = 0.99; Table 2). The mean migration speed of Herring Gulls from the eastern Arctic was 185 ± 127 km/day (*n* = 8), similar to the mean migration speeds of Herring Gulls from Newfoundland (152 ± 178 km/day, *n* = 12), Sable Island (189 ± 83 km/day, *n* = 17) and the Bay of Fundy (167 ± 97 km/day, *n* = 11; Fig. 2f). However long distance migrants did travel at higher speeds on travel days (χ^2^_3_ = 9.61, *p* = 0.02). Birds from the eastern Arctic travelled at 366 ± 107 km/day, while birds from Newfoundland, Sable Island and the Bay of Fundy respectively travelled at 235 ± 155 k/day, 229 ± 62 km/day, and 189 ± 80 km/day (Fig. 2g).

Long distance migrants from the eastern Arctic used an average of 34 ± 26 stopover days during their migrations. The short distance migrants used fewer stopover days (χ^2^_3_ = 14.28, *p* < 0.01; Table 2); birds from Newfoundland stopped for 18 ± 16 days, birds from Sable Island stopped for 5 ± 11 days, and birds from the Bay Fundy stopped for 3 ± 4 days (Fig. 2h). However, the distance travelled per stopover day taken was similar between long and short distance migrants. Gulls from the eastern Arctic travelled 1187 ± 1975 km per stopover day, while gulls from Newfoundland, Sable Island and the Bay of Fundy respectively travelled 465 ± 593 km, 1103 ± 625 km, and 634 ± 473 km per stopover day (Fig. 2i). There was also no significant difference in the length of stopovers taken by long and short distance migrants. Each stopover lasted 12 ± 11 days for eastern Arctic birds, 8 ± 7 days for Newfoundland birds, 5 ± 11 days for Sable Island birds, and 3 ± 4 days for Bay of Fundy birds (Fig. 2j).

## Discussion

Within the four populations we studied, Herring Gulls showed a great deal of individual variation, but in general the migration strategies used by long distance migrants breeding in the eastern Arctic and short distance migrants breeding in Atlantic Canada shared many similarities. Each of the populations we studied generally followed coastal migration routes that were at least 30% longer than a direct course. The overall speeds of their migrations, which encompasses both stopover and travel periods, were not statistically different (Table 2). However, long distance migrants did travel at higher speeds on travel days. Although long distance migrants used more stopover days overall, there was no clear difference between the stopover length or the ratio of distance travelled to stopover days between the short and long distance migrants.

We found that regardless of migration distance, both the Arctic and Atlantic Herring Gulls generally followed indirect migration routes along the Atlantic coast. If long-distance migrants were using an time-minimizing strategy, we had predicted that they would use more direct routes. In contrast, we found that the long distance migrants used less direct routes than the short distance migrants. Herring Gulls appeared to prefer coastal migration routes, despite the fact that they are capable of acquiring food from freshwater habitats and anthropogenic sources like landfills, urban areas, and farm fields [64]. Gulls likely prefer these coastal habitats both because these areas contain predictable sources of food, and because coastal topography creates opportunities for energetically-efficient soaring [27, 65]. Lesser black-backed gulls (*Larus fuscus*) from the Netherlands similarly followed the coast, using indirect routes on their short distance migration [28]. By contrast, lesser black-backed gulls from Norway appear to use more direct routes on their long distance migration [66]. However, it is important to note that this population crosses the Sahara desert, an ecological barrier with scarce food and water resources and a physiologically taxing climate [67]. It may be that habitat and food availability are more important drivers of migration strategy for gulls than migration distance. Furthermore, there is some evidence that gulls may prioritize neither time nor energy expenditure during flight [68].

There was no statistical difference in the overall speed of migration between the populations studied. Compared to the closely related Lesser Black-backed Gull, the overall autumn migration speed of Herring Gulls in eastern North America (152–189 km/day) appears to fall between that of short distance migrants (44 km/day [28];) and long distance migrants (371 km/day [66];). We observed substantial individual variation in overall migration speed within each population, consistent with the behavioural flexibility of Herring Gulls as generalists. Species with more specialized diets may have less individual variation in their migration strategies, which may be attributed to constraints on their foraging and stopover locations [69–71].

Given that overall migration speed was comparable between short and long distance migrants, it is not surprising that there was also not a clear relationship between our stopover metrics and migration distance. A number of recent studies have found that differences in overall migration speed within a population are mostly driven by differences in the extent to which birds use stopovers during migration, while travel behaviour tends to be more consistent [14, 28, 30, 72]. Although Herring Gulls made extensive use of stopovers, individuals mostly did not congregate in population-specific staging areas, but rather individuals stopped throughout their migratory routes. As foraging generalists, large gulls can find food in a diversity of habitats, and are therefore much less restricted in their selection of stopover sites than species with specialized foraging habits such as shorebirds [73].

One case where long distance migrants did appear to have notably different behaviour from short distance migrants was during travel days. Herring Gulls from the eastern Arctic covered greater distances on travel days than gulls from Newfoundland, Sable Island, and the Bay of Fundy (Fig. 2g). However, we note that our measure of travel speed was an estimate of net displacement distance during a day of travel rather than an accurate estimate of their instantaneous speed [46]. It is possible Herring Gulls from the eastern Arctic were truly flying at faster speeds to cover greater distances, but they may also simply be travelling more direct routes to cover the distance more efficiently or travelling more hours per day. These results indicate that there is some differences in migration strategy between long and short distance migrants, suggesting that more information might help clarify what factors are driving their behaviour.

It seems plausible that there may be other more subtle differences in migration strategy between short and long distance migrants that we would have been unable to detect due to our relatively low sample sizes. For instance, there may be a confounding influence of sex [74] or carry-over effects [75]. It is also interesting to note the high amount of explanatory power of individual ID, indicated by the high conditional *R*^2^ values for our models. This pattern suggests that despite flexibility within the population, individual gulls appeared to be consistent in their migratory strategies between years.

## Conclusions

As generalists, Herring Gulls had flexible migration strategies. Herring Gulls use indirect routes, regardless of whether they are short or long distance migrants. They tend to migrate at a moderate overall migration speed with regular stopovers. This impression of their migratory strategy suggests Herring Gulls are not minimizing the duration of their migration. By comparing diverse populations within the same species, our findings add to the list of exceptions to the idea that long distance migrants are necessarily time-limited. We hypothesize that highly flexible generalist species such as Herring Gulls are less likely to be constrained by time when optimizing their migration strategies.

## Data Availability

The datasets supporting the conclusions of this article are available in the Movebank Data Repository: Gilchrist et al. 2020, 10.5441/001/1.1r1s4v8d; Ronconi and Taylor 2020, 10.5441/001/1.3264ss3v; Ronconi and Shlepr 2020, 10.5441/001/1.t5n4s456; Ronconi and Shlepr 2020, 10.5441/001/1.282vr7kd; Fifield et al. 2020, 10.5441/001/1.27244v55; Clark et al. 2020; 10.5441/001/1.3th8b5q3.

## Abbreviations

GLMM: Generalized Linear Mixed Model
GPS: Global Positioning System
MA: Massachusetts, United States of America
MCMC: Monte Carlo Markov Chain
NB: New Brunswick, Canada
NL: Newfoundland, Canada
NS: Nova Scotia, Canada
NU: Nunavut, Canada
PTT: Platform Terminal Transmitter
UHF: Ultra High Frequency
